# Meeting Report: 3^rd ^International Workshop on Insulin & Cancer Heidelberg, Germany, October 30-31, 2010

**DOI:** 10.1186/1758-5996-2-73

**Published:** 2010-12-21

**Authors:** Ernst Chantelau, Doris Mayer

**Affiliations:** 1 Formerly Heinrich-Heine-University, Düsseldorf, Germany; 2 Hormones and Signal Transduction, German Cancer Research Centre Heidelberg, Heidelberg, Germany

## Abstract

The 3rd International Workshop on Insulin & Cancer was held on October 30-31, 2010 at the German Cancer Research Centre in Heidelberg/Germany. The topics followed-up the discussions of the previous workshops: possible differences in mitogenicity between natural insulin and genetically engineered insulin derivatives (insulin analogues), as shown by laboratory studies and epidemiologic studies alike; molecular studies on the links between metabolic and mitogenic effects of insulin, and of hyperinsulinaemia in particular; epidemiologic evidence of interferences between insulin and other hormones, particularly sex hormones, and obesity-associated cancer; the involvement of inflammatory cytokines produced by fat tissue in obesity-associated cancer; aspects of drug-design (binding drugs to albumin) and, last but not least, detection and investigation of circulating cancer cells.

## Report

Since the previous International Workshops on Insulin and Cancer held in Düsseldorf/Germany in 2007 and in 2008 [1], considerable progress in the study of the issue has been noticeable. Hence, the 3rd International Workshop moved to the German Cancer Research Centre in Heidelberg/Germany, attracting 15 speakers and an audience of 40 participants from many countries. Like the previous ones, the 3^rd ^workshop was sponsored entirely by private persons and non-profit organisations, namely the Insulin Dependent Diabetes Trust (IDDT) from Northampton/UK, the Förderverein für Kinder und Jugendliche mit Diabetes mellitus der Universität Köln from Cologne/Germany, and the German Cancer Research Centre (DKFZ).

The sessions started with issues related to the mitogenicity of insulin analogues. With respect to the recent commission by the EMEA "to further investigate the possible increased risk of cancer associated with the use of insulin glargine..." [2], the safety profile of insulin glargine (Lantus^®^) was of particular interest. Kristin Eckardt reviewed the most recent laboratory studies on this topic, including previous own data. Doris Mayer presented her recent investigations of insulin analogues in breast cancer cells showing an increased mitogenicity of Lantus^® ^in cells highly expressing IGF-1 receptors. Haim Werner showed his studies on differences in signalling between long-acting insulin analogues (insulin glargine [Lantus^®^] or insulin detemir [Levemir^®^]) which may be contributing to an increased mitogenicity of these compounds. Together with results from other experimental studies and epidemiological data published recently, these findings seem to underpin the safety concerns, which in May 2010 had led the EMEA to withhold unlimited approval of Lantus^® ^(".... the Marketing Authorization Holder should submit one additional renewal application in 5 years time") [2]. Harald Enzmann, representative of the German regulatory board BfArM and co-author of several cell culture studies on Lantus^®^, presented the regulator's perspective of insulin analogues. He considered the safety record for Lantus^® ^"inconclusive". A commentary from the floor cautioned that Lantus^® ^*in vivo *undergoes biotransformation into lesser mitogenic compounds. This process, however, exhibits considerable inter-individual variability. Thus, subjects with a slow biotransformation and, consequently, a longer incubation time with Lantus^® ^*in vivo*, may be at increased risk for development and progression of malignancies [3]. Such an association may probably remain undetectable by conventional epidemiologic studies. Recently, it was therefore concluded in a paper by Müssig et al., that " studies are warranted to identify and follow patients potentially at risk for cancer because of altered insulin glargine biotransformation" [3].

A study from a regional cancer registry was presented by Hans W. Hense. For the first time in Germany, the regional diabetes treatment registry was linked with the cancer registry, showing an increased incidence of liver cancer, and decreased incidence of breast and prostate cancer, in type-2 diabetic patients. However, the effects of the type of anti-diabetic treatment have as yet not been fully evaluated.

The next sessions dealt with basic science data. Aurelio Teleman reported on his work on insulin signalling in Drosophila in which he found a phosphatase subunit (PPP2R5C) which may be a novel regulator of proliferative and metabolic effects. Boris Draznin focused on the fact that insulin- by increasing the amount of farnesylated Ras molecules- enhances the effects of growth factors such as IGF-1, and that hyperinsulinaemia thereby augments the mitogenic effects of growth factors (e.g. on tumour cells). Stephan Herzig alluded to chronic aberrant inflammatory signalling, and transcription factor functions, involved in metabolic and mitogenic risks seen in obese patients with type-2 diabetes mellitus. Jean Grisouard discussed AMP-dependent kinase (AMPK) affecting the production of adipokines (e.g. TNF alpha, IL-6, leptin), which increase insulin resistance of adipocytes, whereas Metformin activating AMPK, reduced (pro-inflammatory) IL-6 and increased IL-1 receptor antagonist (which is anti-inflammatory), thereby potentially inhibiting IL-6 associated insulin-resistance and IL-1 induced tumour progression. These data were largely corroborated by John Reynolds in a later session: he found evidence for upregulated inflammatory cytokines in the fat tissue of his patients operated for oesophageal carcinoma.

By a kind of mirror-image, Zvi Laron reported a follow-up of his earlier investigation on subjects with congenital IGF-1 deficiency, observing no malignancies in more than 500 homozygous patients, whereas various malignancies were found in their heterozygous family members.

Also Rudolf Kaaks showed a follow-up of his earlier epidemiologic data, now focusing on the impact of insulin on sex hormones and SHBG (sexual hormone binding globulin), which might explain some of the association between obesity and cancer incidence and proliferation.

As one of the last speakers, Eva Frei explained how albumin can be used to transfer and accumulate drugs or other compounds into tumour tissues; concerning the binding of insulin detemir (Levemir^®^) to albumin after subcutaneous injection, a paucity of data was noted in this respect. Burkhard Brandt reported on his experiences with the extraction and investigation of circulating tumour cells using antibodies coupled to nanobeads.

Taken together, considerable progress in the study of the Insulin-Cancer-Connection was noted, making a continuation of the International Workshops on Insulin & Cancer desirable. Meanwhile, the proceedings of the 3^rd ^workshop will hopefully be published in one of the forthcoming issues of "Diabetology & Metabolic Syndrome" in 2011.

## Competing interests

The authors declare that they have no competing interests.

## Authors' contributions

EC drafted the report, DM participated in the writing of the final manuscript. All authors have read and approved the final version of the article.
